# Phosphorylation of a Tumor-Derived ASXL2 Epitope Remodels the HLA-Bound Peptide Conformational Ensemble and Interaction Network of the Peptide–HLA Complex

**DOI:** 10.34133/csbj.0176

**Published:** 2026-07-23

**Authors:** Jiahui Zhang, Lin Lv, Bairun Chen, Xinpei Yi

**Affiliations:** ^1^ National Facility for Protein Science in Shanghai, Shanghai Advanced Research Institute, Chinese Academy of Sciences, Shanghai 201210, China.; ^2^ University of Chinese Academy of Sciences, Beijing 100049, China.; ^3^ School of Public Health, Fudan University, Shanghai 200032, China.; ^4^ Department of Bioinformatics and Biostatistics, School of Life Sciences and Biotechnology, Shanghai Jiao Tong University, Shanghai 200240, China.

## Abstract

The expansion of cancer immunotherapy has made it increasingly important to identify tumor-specific human leukocyte antigen (HLA) class I epitopes and to define the molecular determinants of peptide–HLA binding and stability. Posttranslationally modified epitopes, particularly phosphorylation, can reflect oncogenic signaling states and offer highly tumor-selective targets. Despite their potential, the structural and biophysical mechanisms by which phosphorylation reshapes the conformational ensemble and interaction network of peptide–HLA complexes remain unclear. Here, we investigate a phosphorylated ASXL transcriptional regulator 2 (ASXL2)-derived epitope supported by confident tandem mass spectrometry-based phosphosite localization and detected across multiple cancer types. We performed all-atom molecular dynamics simulations of peptide–HLA complexes to compare the phosphorylated peptide, its nonphosphorylated counterpart and its protonated-phosphorylated state. Our simulations indicate that phosphorylation reconfigures the noncovalent interaction network within the binding groove by introducing new local contacts. Principal component analysis further revealed that phosphorylation restricts peptide conformational fluctuations, thereby altering the conformational ensemble of the peptide–HLA complex. Furthermore, protonation of the phosphorylated peptide remodels the noncovalent interaction network and conformational ensemble. Together, these findings provide a structural and biophysical basis for how phosphorylation can modulate the conformational ensemble and interaction network of peptide–HLA complexes, which may help prioritize cancer-specific targets for immunotherapeutic development.

## Highlights

• An MS/MS-supported ASXL2 phospho-peptide–HLA-A*31:01 complex was modeled.
• Phosphorylation reshapes local and distal peptide–HLA interaction networks.
• Phosphorylation restricts the HLA-bound peptide conformational ensemble.
• Phosphate protonation remodels peptide–HLA contacts and conformational sampling.

## Graphical Abstract

**Figure:**
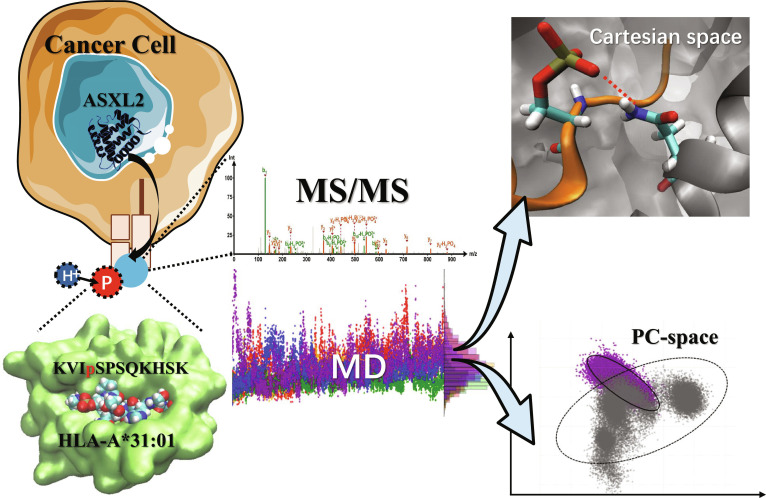

## Introduction

The presentation of antigenic peptides by human leukocyte antigen (HLA) molecules is the crucial first step in triggering adaptive immune recognition [1,2]. Intracellularly, proteins are continuously processed into short peptides [3], a specific subset of which is loaded into the binding groove of HLA class I molecules and presented on the cell surface as peptide–HLA complexes [4]. Circulating T cells continuously survey these peptide–HLA complexes via their T cell receptors (TCRs) [5]. Recognition of a nonself or aberrant peptide triggers robust T cell activation and downstream effector responses aimed at eliminating the presenting cell, a mechanism central to tumor clearance [6]. Consequently, deciphering the exact properties of peptide–HLA interactions is paramount to cancer immunotherapy [7,8], serving as the structural blueprint for the rational design of therapeutic vaccines [9], engineered TCRs [10], and TCR-mimic antibodies [11].

Beyond canonical targets, posttranslationally modified (PTM) antigenic peptides introduce a biologically informative layer to the peptide–HLA landscape. In malignancies, aberrant signaling and metabolism produce a unique repertoire of modified peptides, most notably through phosphorylation, acetylation, and methylation. These PTM neoantigens represent a highly substantial class of therapeutic targets: They not only offer enhanced tumor selectivity but can also be shared across diverse patient populations, eliciting robust immunogenicity [12]. Despite their immense clinical potential, these modified antigens have been largely overlooked by previous studies. This historical neglect stems from a major technical bottleneck: Because conventional genomic and transcriptomic approaches cannot capture these dynamic posttranslational events, PTM antigens can exclusively be detected through proteomics-level mass spectrometry. Today, tandem mass spectrometry (MS/MS) within immunopeptidomics has become indispensable, providing the only direct, site-resolved evidence for PTM identity and localization [13], thereby enabling the confident discovery of these elusive targets.

Yet, successful mass spectrometry identification addresses only part of the challenge; interpreting the therapeutic potential of these PTM neoantigens requires a deep understanding of their binding biophysics [14]. Recently, some researchers have begun to investigate how modifications such as phosphorylation affect peptide–HLA interactions, developing tools to assess the binding affinity and static structures of phosphorylated peptide–HLA complexes [15–17]. However, these investigations have largely focused on prediction or static structural models, leaving ensemble-level conformational behavior less well characterized. Characterizing the interaction networks of peptide–HLA complexes at atomic resolution is of substantial scientific importance, as it not only reveals detailed ensemble-level structural insights into the peptide–HLA complex of interest but also provides a key basis for understanding the biophysical mechanisms underlying the binding process. Furthermore, the vast majority of current predictive models and tools remain optimized almost exclusively for unmodified canonical sequences [18,19], which might inhibit the clinical research of valuable PTM cancer antigens.

**Fig. 1.**
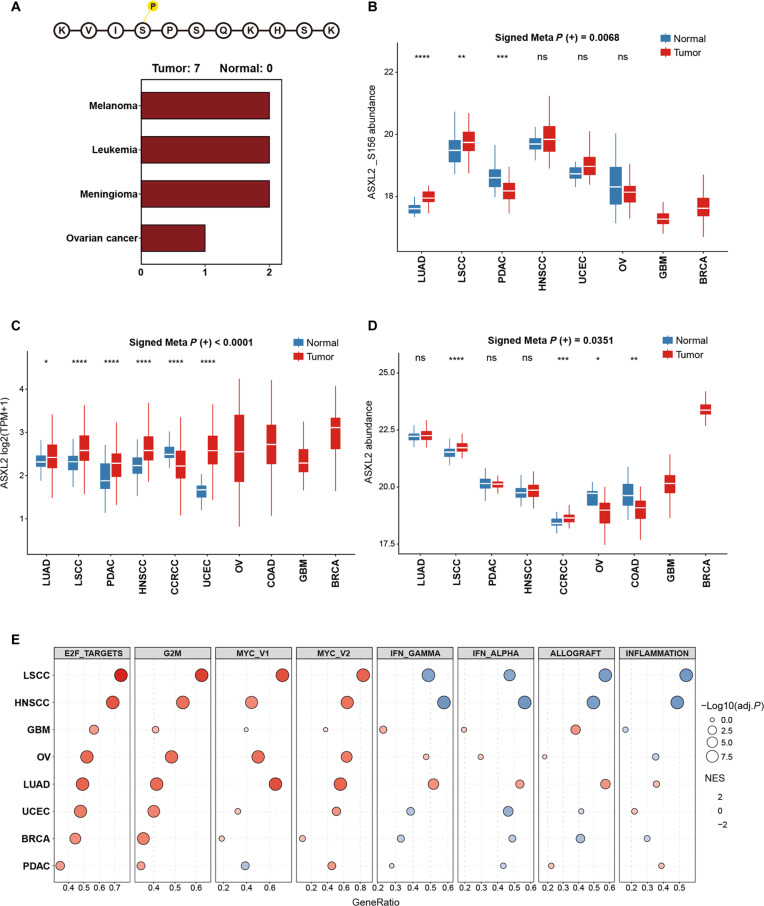
Identification and multiomics characterization of the tumor-specific ASXL transcriptional regulator 2 (ASXL2) phospho-peptide. (A) Schematic sequence (top) and detection frequency statistics in the caAtlas database (bottom) of the ASXL2 phospho-peptide. (B to D) Pan-cancer dysregulation of ASXL2 across multiple omics layers. Boxplots compare the abundance of (B) the specific S156 phosphosite, (C) mRNA expression, and (D) total protein levels between tumor (red) and normal (blue) tissues in the Clinical Proteomic Tumor Analysis Consortium dataset. The signed Meta *P* value indicates the global statistical significance of the up-regulation in tumors. (E) Functional characterization of ASXL2 phosphorylation via gene set enrichment analysis. ns, not significant; *P < 0.05; **P < 0.01; ***P < 0.001; ****P < 0.0001. LUAD, lung adenocarcinoma; LSCC, lung squamous cell carcinoma; PDAC, pancreatic ductal adenocarcinoma; HNSCC, head and neck squamous cell carcinoma; CCRCC, clear cell renal cell carcinoma; UCEC, uterine corpus endometrial carcinoma; OV, ovarian serous cystadenocarcinoma; COAD, colon adenocarcinoma; GBM, glioblastoma multiforme; BRCA, breast invasive carcinoma; NES, normalized enrichment score.

To address this critical gap, we report a case study of a PTM peptide–HLA complex. We focus on a specific phosphorylated antigenic peptide, KVIpSPSQKHSK, from ASXL transcriptional regulator 2 (ASXL2) (Fig. 1A, top, and Fig. S1) [20]. ASXL2 is an epigenetic regulator with multiple functions [21]. Moreover, emerging evidence underscores its clinical significance in various malignancies, where its aberrant expression is associated with tumorigenesis and poor prognosis in colorectal and pancreatic cancers, as well as immunomodulation in head and neck squamous cell carcinoma [22–34]. Interestingly, Fig. 1A (bottom) shows that this peptide was detected only in 7 tumor samples in the caAtlas database [25] but was completely absent in normal tissues. This tumor-restricted detection pattern within caAtlas spans multiple cancer types, including melanoma, leukemia, meningioma, and ovarian cancer, as supported by MS/MS data. Preliminary pan-cancer analysis leveraging the Clinical Proteomic Tumor Analysis Consortium (CPTAC) dataset [26,27] reveals that ASXL2 is substantially dysregulated across multiple omics layers, including mRNA expression, protein abundance, and site-specific phosphorylation (Fig. 1B to D). These consistent alterations between tumor and normal tissues underscore the potential clinical relevance of this antigen and its posttranslational modifications. Furthermore, our pan-cancer pathway analysis reveals a distinct pattern where this phosphorylation correlates with up-regulated tumorigenic signatures and dampened immune surveillance pathways (Fig. 1E). These correlations suggest a possible association between ASXL2 phosphorylation and tumor-promoting or immune-suppressive pathway signatures.

Although experiments can reveal the biophysical properties of such peptide–HLA systems [28], molecular dynamics (MD) simulations [29] can provide detailed atomic-scale insights into the conformational behavior of such systems, as reported in previous works [30–35]. In this work, we performed all-atom MD simulations of the MS/MS-validated ASXL2-derived peptide bound to HLA-A*31:01 using the AMBER phosaa19SB force field for phosphorylated residues together with the ff19SB protein force field [36,37], following previous MD studies that adopted related force-field frameworks in experimentally supported molecular simulations [38,39]. As described in Materials and Methods, trajectories were generated for the phosphorylated, nonphosphorylated, and protonated-phosphorylated peptide–HLA systems. We first examined the phosphorylation-induced changes in local and global interaction networks, after which we performed principal component analysis (PCA) to reveal the differences among the conformational ensembles of the systems of interest. We then investigated the effect of the protonation of the phosphate group, i.e., how protonation alters the interaction networks and conformational ensembles of the phosphorylated peptide–HLA complex. Finally, we provide a plausible theoretical interpretation of the phosphorylation-induced conformational fluctuation changes and suggest that ensemble-level conformational information may help guide future validation, screening, and design of TCRs targeting PTM peptide–HLA complexes.

## Materials and Methods

### Computational materials and data sources

We selected a PTM antigen derived from ASXL2 (phosphorylation) from the caAtlas database [25] and focused on the HLA allele HLA-A*31:01. For comparison, we constructed both phosphorylated and nonphosphorylated peptide–HLA complexes. For the phosphorylated complex, we additionally modeled a protonated state to assess how protonation affects the conformational ensemble and interaction network.

The HLA-A*31:01 sequence was obtained from the Immuno Polymorphism Database–International ImMunoGeneTics/Human Leukocyte Antigen Database [40]. The mature β2-microglobulin sequence was taken from the FASTA file of Protein Data Bank ID: 1AO7 [41] as provided by the Research Collaboratory for Structural Bioinformatics Protein Data Bank website. For computational efficiency, only the folded extracellular HLA-A*31:01 α-chain region, corresponding to residues Gly25 to Glu299, was retained for MD simulations. To avoid artificial edge charge effects, the truncated protein was capped with an ACE residue at the N-terminus and an NME residue at the C-terminus of the α-heavy chain.

### Data acquisition and statistical analysis

Harmonized CPTAC proteomic and open-access genomic data were obtained via the Proteomic Data Commons (https://pdc.cancer.gov/pdc/cptac-pancancer). Raw genomic and transcriptomic data were accessed via the Genomic Data Commons Data Portal (https://portal.gdc.cancer.gov; Database of Genotypes and Phenotypes Study Accession: phs001287.v16.p6). Controlled datasets hosted in the Cancer Data Service were accessed through National Cancer Institute Data Access Committee-approved, Database of Genotypes and Phenotypes-compiled whitelists.

### Differential expression and pathway enrichment analysis

For statistical analysis, differential abundance between tumor and normal tissues was assessed using the Wilcoxon rank-sum test. Pan-cancer significance was evaluated by a signed meta-analysis using Stouffer’s *z*-score method to derive a combined Meta *P* value. Subsequently, to explore the biological signaling pathways associated with ASXL2 phosphorylation, we performed gene set enrichment analysis. Leveraging the comprehensive proteomic data from the CPTAC dataset, tumor samples were stratified into “High” and “Low” groups based on the median abundance of the specific ASXL2 phosphosite (S156). Differential protein expression analysis was performed between the High and Low phosphorylation groups to generate a ranked gene list (using t-statistics). Gene set enrichment analysis was then conducted using the MSigDB Hallmark gene set collection via the clusterProfiler R package.

### Simulation setup and MD protocol

We used AlphaFold3-predicted models as starting structures for geometry optimization [16]. In the AlphaFold3 input, phosphorylated serine was specified using the residue code SEP. All sequences used for structure prediction are provided in Supplementary File 1. Each complex was solvated in a truncated octahedral TIP3P water box [42], with a minimum buffer of 10 Å from the peptide–HLA complex to the box boundary, and 0.15 M NaCl was added to the solvent. We used AMBER phosaa19SB [36] and ff19SB force fields [37] in this work.

All energy minimization, equilibration, and production MD simulations were performed with AMBER 24 [43]. The protocol was as follows. First, we restrained the peptide–HLA complex and ions and minimized only the solvent; we then released restraints and performed a second minimization of the full system. Next, we heated the system from 0 K to 310 K under NVT conditions, followed by 1 ns of NVT equilibration and 1 ns of NPT equilibration. The final coordinates from the NPT stage were used to initialize production simulations. For each peptide–HLA system, we ran 5 parallel 550-ns production simulations, of which only the last 500 ns were used for analysis. For peptide-only systems, we performed 3 parallel 110-ns production simulations, and only the last 100 ns were used for analysis. A 10-Å cutoff was applied to the nonbonded interactions, and the particle mesh Ewald method [44] was used to calculate the electrostatic interactions with cubic-spline interpolation and a grid spacing of approximately 1 Å.

### Analysis of ligand noncovalent interactions and conformational ensembles

Hydrogen bonds, salt bridges, and hydrophobic contacts were quantified using MDTraj [45]. Backbone Cα root-mean-square deviation (RMSD), Cα root-mean-square fluctuation (RMSF), and PCA were computed with MDTraj [45]. Hydrogen bonds were defined using a 0.25-nm H-A distance cutoff and a 120° D-H-A angle cutoff, salt bridges using a 0.40-nm charged-atom distance cutoff, and hydrophobic contacts using a 0.45-nm side-chain carbon–carbon distance cutoff. PCA [46] was performed after structural alignment to a common reference, using Cα atoms of the selected component. The PCs were derived from the cartesian coordinates of Cα atoms for each amino acid residue, and the PCs of the 2 compared systems were computed from concatenated trajectories to enable direct comparison.

### Free energy calculations

Free energies and binding free energy changes were estimated using both the generalized Born and Poisson–Boltzmann approaches with MMPBSA.py in AMBER 24 [43]. For Poisson–Boltzmann calculations, the dielectric constant was set to 2 for the solute interior and 80 for the solvent. For each MD run, 1,000 frames were extracted at evenly spaced time points from the production trajectory for free energy analysis. For each system, the mean free energy value from each independent MD trajectory was used as the statistical unit, rather than treating individual frames as independent observations. Pairwise comparisons between systems were performed using 2-sided Welch’s *t* test.

## Results

### Phosphorylation alters noncovalent interactions locally and globally

Both the phosphorylated and nonphosphorylated AlphaFold3 models showed high prediction confidence, with comparable pTM values of 0.81 and 0.80, ipTM values of 0.94 and 0.95, mean pLDDT values of 85.01 and 85.05, and low chain-pair minimum predicted aligned error values of 0.76 to 2.17 Å and 0.76 to 2.62 Å, respectively (detailed reports are provided as Supplementary File 2). To verify the appropriateness of our simulations, we first checked the time-series RMSD and per-residue RMSF of all the production trajectories we generated in this work, as shown in Figs. S2 and S3. Across all simulations, RMSD values remained within a narrow range, indicating that the systems remained stable during the production window. The RMSF profiles showed elevated thermal fluctuations primarily at the protein termini, whereas nonterminal residues showed lower fluctuations, which is expected. Together, these analyses support that our simulation protocol is robust and suitable for the subsequent analyses.

**Fig. 2.**
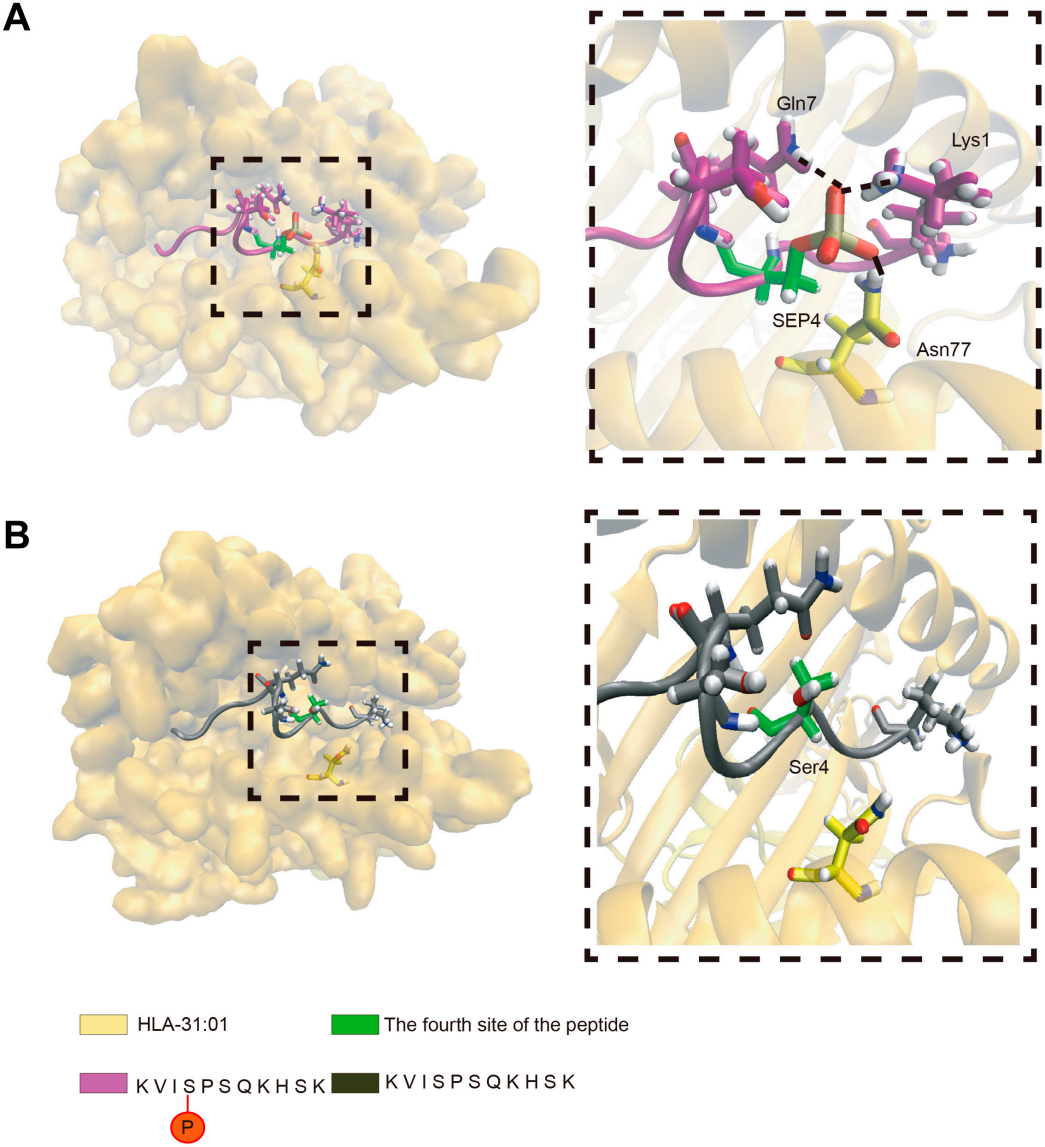
Overall and zoomed-in views of the peptide–human leukocyte antigen (HLA) complexes. (A) The phosphorylated complex. (B) The nonphosphorylated complex. Noncovalent interactions are shown as dashed lines, and interacting residues are labeled. The fourth peptide residue, which is phosphorylated in the phosphorylated peptide, is colored green.

The local environments around the focal site in the phosphorylated and nonphosphorylated systems are shown in Fig. 2. In the enlarged views, the phosphorylated complex exhibits a denser interaction pattern, suggesting that phosphorylation promotes additional contacts. Specifically, phosphoserine at position 4 (SEP4) forms interactions with Lys1 and with Gln7 within the peptide, and it also forms a phosphorylation-dependent contact with Asn77 of the HLA molecule. These observations indicate that phosphorylation remodels the local nonbonded interaction network around the modified site by introducing new contacts. In subsequent analyses, we found that the newly formed intrapeptide interactions are stronger than the newly formed peptide–HLA interactions, consistent with a more rigid peptide conformation as described in the following section. This increased rigidity may facilitate stable binding within the HLA groove.

**Fig. 3.**
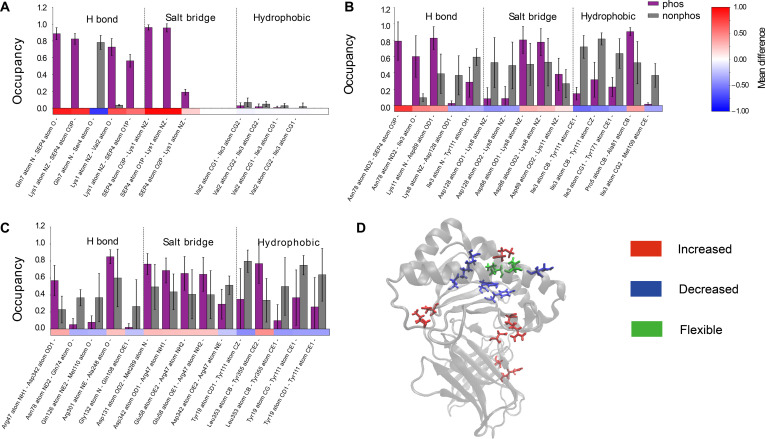
Noncovalent interaction differences between phosphorylated and nonphosphorylated peptide–human leukocyte antigen (HLA) complexes. (A) Peptide–peptide contacts, (B) peptide–HLA contacts, and (C) HLA–HLA contacts. The color bar below each panel indicates the mean occupancy difference. (D) Residues involved in the top increased interactions are shown in red, residues involved in the top decreased interactions are shown in blue, and residues involved in both increased and decreased interactions are shown in green.

Beyond the local changes around the phosphorylated site, phosphorylation also reshapes interaction networks across the peptide–HLA complex. As shown in Fig. 3, peptide–peptide, peptide–HLA, and HLA–HLA interactions differ substantially between the phosphorylated and nonphosphorylated systems, with some of the largest changes occurring at residues distant from the modified position. To visualize these effects, the top 3 increased and decreased peptide–HLA and HLA–HLA interactions are highlighted in Fig. 3D. We further observed that the freely solvated phosphorylated peptide has more noncovalent interactions than its nonphosphorylated counterpart, as shown in Fig. S4. Many of these interactions are shared by the HLA-bound phosphorylated peptide, which indicates that phosphorylation may preorganize the peptide toward an HLA-bound-like state. However, despite the global alteration of the interaction network, our free energy estimates did not provide a statistically robust ranking of the relative binding affinities among the phosphorylated, protonated-phosphorylated, and nonphosphorylated peptides (Fig. S5).

### Phosphorylation stabilizes the ligand conformational ensemble in the peptide–HLA complex

**Fig. 4.**
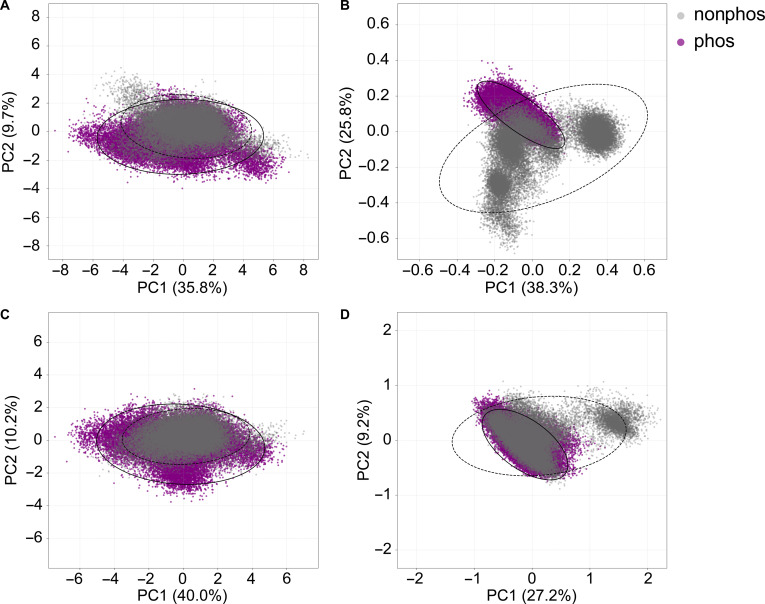
Principal component analysis of backbone conformational ensembles for the phosphorylated and nonphosphorylated peptide–human leukocyte antigen (HLA) systems, projected onto the same reference frame. (A) Whole complex, (B) peptide, (C) HLA heavy α-chain, and (D) β2-microglobulin. Solid and dashed contours denote the 95% confidence ellipses for the 2 compared systems, as indicated in the figure legend. The fraction of variance explained by each principal component (PC) is shown in parentheses.

To compare backbone conformational ensembles between the phosphorylated and nonphosphorylated systems, we performed PCA [46], a dimensionality-reduction approach that projects conformational fluctuations onto a small number of collective modes. A more compact PC distribution may indicate a more restricted and stabilized conformational ensemble [47,48]. We focused on the first 2 principal components (PCs), and the results are shown in Fig. 4. We carried out 4 separate PCA analyses for (a) the entire complex, (b) the peptide alone, (c) the HLA heavy chain (α-chain) alone, and (d) β2-microglobulin alone.

From the PCA results, phosphorylation appears to increase the conformational heterogeneity of the overall complex, particularly the HLA heavy chain (α-chain), as indicated by a broader distribution of conformations in PC space. In addition, phosphorylation substantially reshapes the dominant backbone conformational modes of both the peptide and β2-microglobulin, consistent with a pronounced shift in their sampled conformational states.

**Fig. 5.**
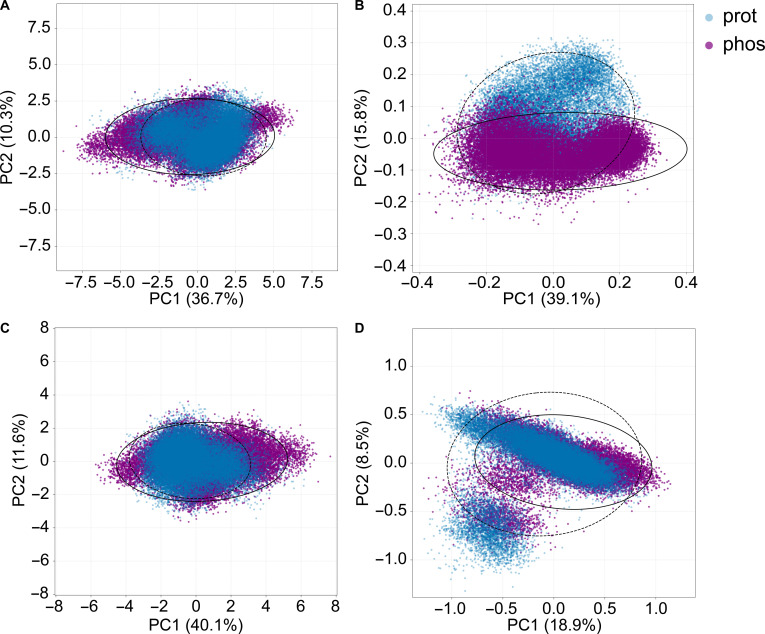
Principal component analysis of backbone conformational ensembles for the protonated-phosphorylated and nonprotonated-phosphorylated peptide–human leukocyte antigen (HLA) systems, projected onto the same reference frame. (A) Whole complex, (B) peptide, (C) HLA heavy α-chain, and (D) β2-microglobulin. Solid and dashed contours denote the 95% confidence ellipses for the 2 compared systems, as indicated in the figure legend. The fraction of variance explained by each principal component (PC) is shown in parentheses.

To quantify changes in the conformational ensemble, we calculated dimensionless PC-space dispersion (*S*) and its change (Δ*S*) using Eqs. (1) and (2). PC-space dispersion was estimated from the first 2 PCs. The fraction of variance explained by PC1 and PC2 is shown in Figs. 4 and 5 for each analyzed component. The probability of each PC-map bin was defined as the number of points in the bin divided by the total number of points for the corresponding peptide–HLA system, with ${\mathrm{\Sigma P}}_{\mathrm{bin}}$ = 1. A bin size of 0.1 × 0.1 PC-score units was used for estimation while an ε value of 10^−6^ was used to avoid numerical divergence.

S=−∑binsPbinlnPbin+ε (1)

∆S=S−Sref (2)

In the phosphorylation comparison, Δ*S* was calculated as $S_{\text{phos}}-S_{\text{nonphos}}$. In the protonation comparison, ΔS was calculated as $S_{\text{prot}}-S_{\text{phos}}$ to quantify the extent to which protonation offsets the phosphorylation-associated dispersion pattern. The calculations indicate that phosphorylation yields a ΔS of −1.02 for the peptide alone and 0.34 for the full complex, indicating a more compact peptide conformational ensemble but a broader conformational distribution of the full peptide–HLA complex; values for the other components are reported in Table 1. Together, these results suggest that phosphorylation locally restricts the HLA-bound peptide within the groove, while increasing the conformational heterogeneity of the full peptide–HLA complex, likely through redistribution of fluctuations to surrounding HLA regions.

**Table 1.** Detailed PCA-derived conformational heterogeneity results

PC-space dispersion	Phos (phos vs. nonphos)	Nonphos (phos vs. nonphos)	Δ*S* = *S*_phos_ − *S*_nonphos_	Phos (prot vs. phos)	Prot (prot vs. phos)	Δ*S* = S_prot_ − *S*_phos_
All	8.00	7.66	0.34	8.03	7.73	−0.30
Peptide	2.21	3.23	−1.02	2.54	2.81	0.26
α-Chain	7.86	7.36	0.51	7.86	7.526	−0.35
β2-Microglobulin	4.63	5.09	−0.46	4.31	4.45	0.13

PCA, principal component analysis; PC, principal component

### Phosphate protonation remodels the interaction network and conformational ensemble of the phosphorylated peptide–HLA complex

Tumor tissues are often characterized by an acidic extracellular microenvironment, largely associated with altered cancer metabolism, lactate accumulation, and limited perfusion [49,50]. Because the protonation state of phosphate groups is pH-dependent, acidic conditions may increase the population of protonated phosphate species, including protonated phosphoserine-like groups [51,52]. Therefore, in addition to the deprotonated-phosphorylated peptide, we also considered the protonated-phosphorylated state to examine whether acidification-related protonation could alter the behavior of the phosphorylated peptide–HLA complex.

As shown in Fig. 5, protonation markedly altered the conformational ensemble of the phosphorylated peptide–HLA complex. Protonation partially reversed the phosphorylation-associated PC-space dispersion pattern, as reflected by the opposite signs of Δ*S* in the protonated-phosphorylated versus phosphorylated comparison. The same trend is supported by the quantitative PCA-derived dispersion metrics summarized in Table 1. These results suggest that protonation partially offsets the conformational stabilization of the peptide introduced by phosphorylation, even though the global peptide–HLA complex becomes less dispersed. Consistent with this interpretation, Fig. S6 further shows that protonation also remodels the interaction network relative to the phosphorylated peptide–HLA complex, indicating that reducing the phosphate charge affects not only local phosphate-mediated contacts but also the broader organization of peptide–HLA interactions.

## Discussion

In this study, we performed MD simulations and trajectory analysis using a protocol whose stability was assessed by replicate RMSD and RMSF analyses. The simulations indicate that phosphorylation of the serine at position 4 in the ASXL2-derived peptide substantially alters both the compactness of the ligand conformational ensemble and the interaction network of the peptide–HLA complex. In this section, we discuss our main conclusions with a plausible theoretical interpretation and discuss possible implications for the design and engineering of immunotherapeutic molecules.

The endpoint free energy calculations were not sufficient to rank the binding affinities of the phosphorylated, protonated, and unmodified peptides. This indicates that the endpoint free energy estimates could not distinguish the relative binding preferences among these peptide states, which might be a consequence of limited statistical power or limitations of free energy estimation algorithms [53–55]. Instead, we focused on the conformational and interaction analyses, which gave a more consistent picture. PCA showed that phosphorylation stabilizes the peptide conformational ensemble, while the interaction analysis showed that the free phosphorylated peptide forms more bound-state-like contacts than the unmodified peptide. This preorganized, binding-competent ensemble might reduce the conformational search required at the early stage of HLA engagement and is consistent with a conformational selection-like contribution to productive binding [56–58]. Thus, although the endpoint free energy calculations were inconclusive, the PCA and interaction data provide a plausible kinetic hypothesis: Phosphorylation may preorganize the peptide toward a more binding-competent ensemble, reducing the conformational search required during HLA engagement rather than necessarily increasing binding affinity in a thermodynamic sense.

A simple electrostatic estimate suggests that the observed interaction and conformational changes cannot be explained solely by the direct long-range field of the phosphate group. If phosphorylation is approximated as a local charge perturbation of 2 elementary charges, the electric field at a distal region of the peptide–HLA complex is:

Er∼2e4πϵ0ϵrr2 (3)

For a distance *r* on the order of 10 nm and an effective relative dielectric constant ε*r* on the order of 1 to 2, this gives a distal field on the order of 10^6^ to 10^7^ V·m^−1^. The energetic bias on a unit charge displaced by a typical atomic-scale distance (~0.1 nm) and a typical atomic charge (~1 elementary charge) is therefore:

∆UfarkBT∼qErδrkBT≪1 (4)

Thus, at distal sites, the direct coulombic bias is expected to be much smaller than thermal energy at 310 K. This argues against a purely long-range electrostatic-field explanation. Instead, phosphorylation is more likely to act through local charge and dipole rearrangement within the peptide-binding groove, followed by propagation through hydrogen-bond, salt-bridge, and hydrophobic packing networks. The conformational response can be interpreted using a linear-response-like ensemble reweighting picture [59]. In this framework, phosphorylation produces opposite covariance patterns for the α-chain and β2-microglobulin: negative for the α-chain and positive for β2-microglobulin. A negative covariance means that large-amplitude conformations are energetically favored after phosphorylation, become more populated, and thus broaden the conformational distribution. This likely applies to the HLA α-chain, which directly contacts the phosphorylated peptide and can accommodate the phosphate group through alternative salt-bridge, hydrogen-bonding, and water-mediated interactions. In contrast, a positive covariance means that large-amplitude conformations are penalized and suppressed, leading to a narrowed conformational distribution. This is consistent with β2-microglobulin, which is distal from the phosphorylation site; its larger fluctuations are unlikely to assist phosphate accommodation and may instead conflict with the remodeled α-chain scaffold, narrowing its conformational ensemble.

As shown in Fig. 4, the phosphorylated and nonphosphorylated systems sampled distinct regions of conformational space, especially at the peptide segment exposed for TCR recognition. This observation suggests that phosphorylation might affect TCR compatibility not only by changing local charge and steric features but also by shifting the ensemble of peptide-presenting states. Given that peptide-dependent tuning of HLA dynamics and ligand-induced changes in TCR recognition have been reported previously [60,61], a TCR selected against the nonphosphorylated peptide–HLA may not necessarily recognize the phosphorylated ensemble with the same efficiency, even when peptide–HLA binding is not substantially weakened. This interpretation remains suggestive and should be viewed as a testable hypothesis rather than direct evidence for altered TCR recognition or immune escape. Future validation would require quantitative immunopeptidomics to compare phosphorylated and nonphosphorylated peptide presentation, peptide–HLA stability or tetramer-stability assays to evaluate complex stability, TCR or TCR-mimic binding assays to measure recognition, and phospho-specific T cell response assays to test functional immunogenicity. Candidate TCRs or phosphorylation-specific peptide–HLA binders may therefore need to be evaluated against multiple representative conformations rather than a single static peptide–HLA model. Recent advances in computational immunopeptidomics, epitope and binder design, TCR-pMHC binding prediction, TCR-pMHC structural modeling, and peptide-MHC-I binder design provide useful methodological context for such ensemble-aware screening [62–69]. Our results should not be viewed as a direct design rule. Instead, they provide a descriptive structural observation that phosphorylation-dependent conformational ensembles may be worth considering in future TCR screening and phosphorylation-specific binder development.

## Data Availability

All data needed to evaluate the conclusions of this study are provided in the article and its Supplementary Materials. The structural models, molecular dynamics simulation input files, analysis scripts, and source data underlying the figures are available through GitHub at: https://github.com/YiCITI/pASXL2_TumorAntigenMD. The code is distributed under the MIT License. Owing to their large file sizes, the complete molecular dynamics trajectories have not been deposited in a public repository but are available from the corresponding author upon reasonable request. No additional restrictions apply to data availability.
