# Supplementary material for: Phosphorylation of a Tumor-Derived ASXL2 Epitope Remodels the HLA-Bound Peptide Conformational Ensemble and Interaction Network of the Peptide–HLA Complex
Source: Comput Struct Biotechnol J. 2026 Jul 23;35(1):0176. doi: 10.34133/csbj.0176 (PMC13392290; doi:10.34133/csbj.0176)
Supplement: Supplementary 1 — Figs. S1 to S6 Supplementary Files 1 to 3 [file csbj.0176.f1.zip › Supplemental Figures_20260703.docx]

**Supplemental Figures**


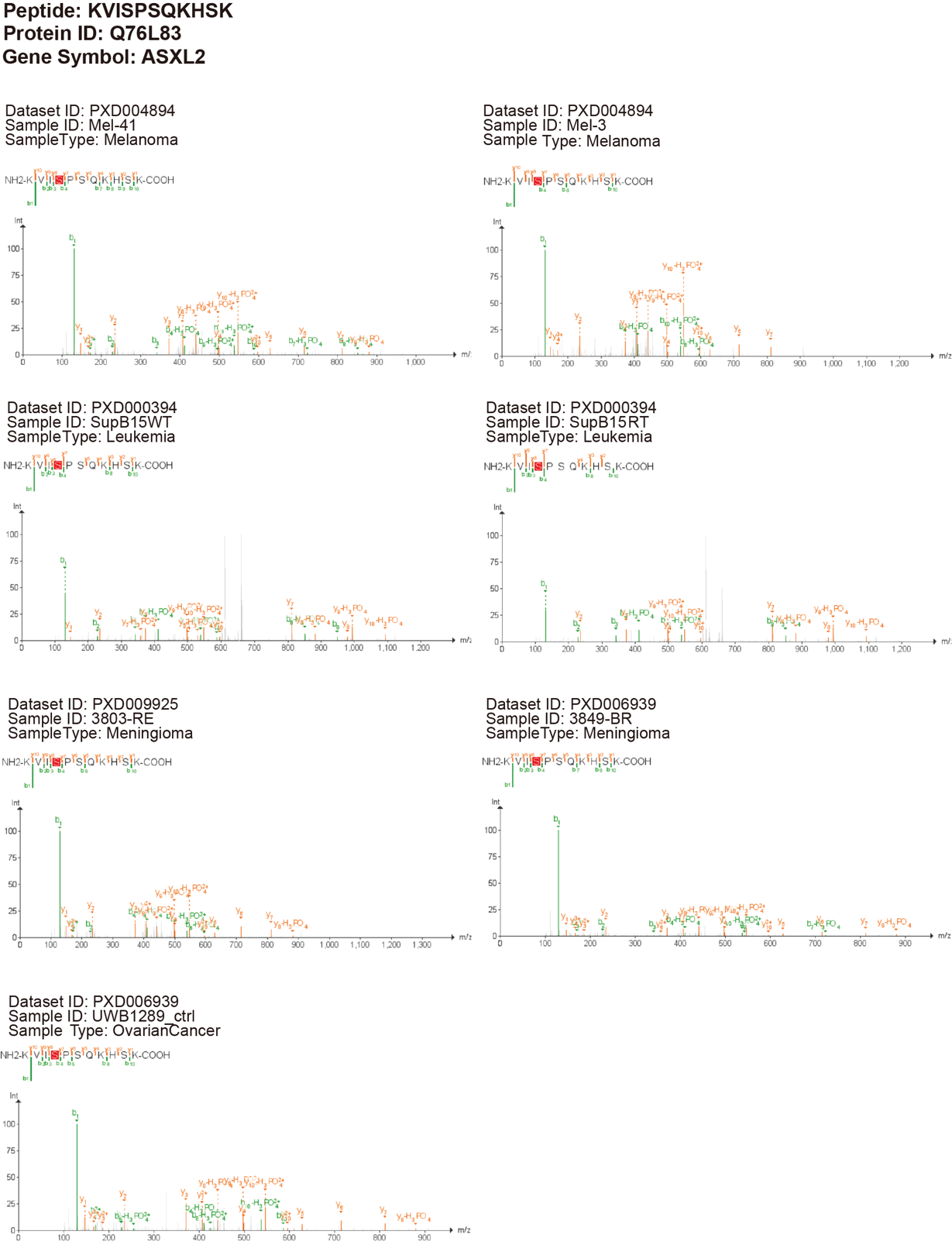


**Supplementary Figure 1. Annotated MS/MS spectra of the ASXL2-derived phospho-peptide retrieved from caAtlas.** Representative spectral plots validating the identification of the KVIpSPSQKHSK sequence in the tumor samples shown in Figure 1A. Dataset IDs and specific cancer subtypes are labeled above each spectrum.


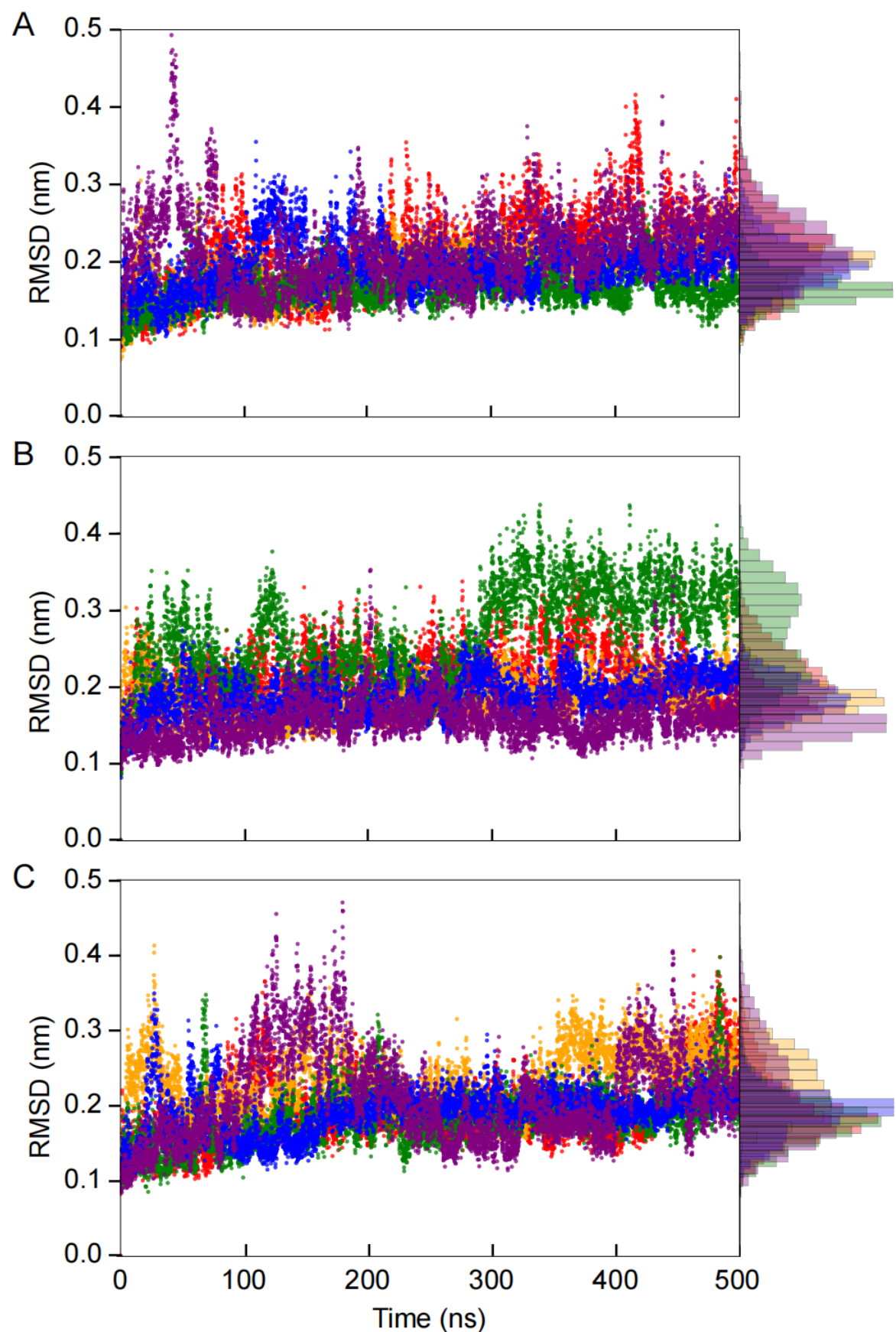


**Supplementary Figure 2. RMSD versus time for all production simulations.** (A) The phosphorylated system, (B) protonated-phosphorylated system and (C) non-phosphorylated system. Each color represents an independent production trajectory.


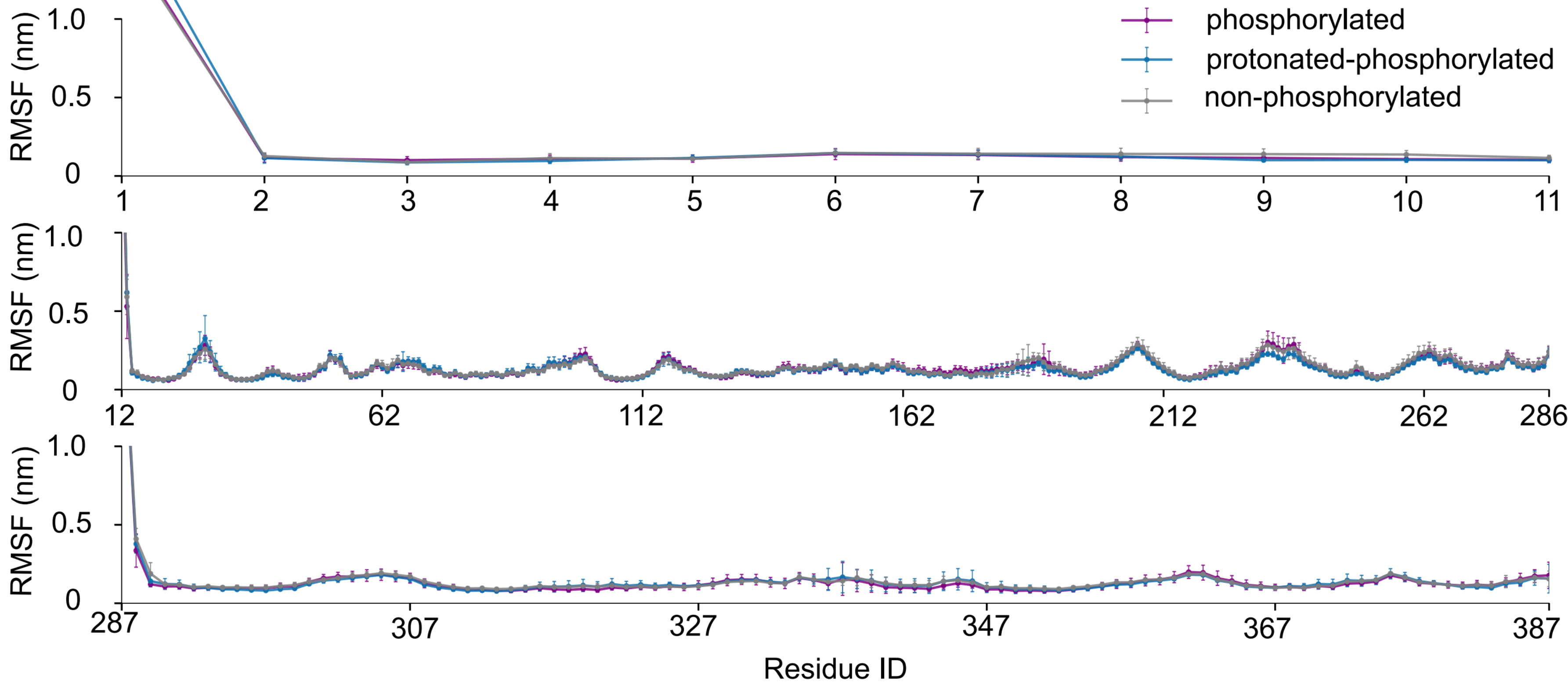


**Supplementary Figure 3. Per-residue RMSF for all production simulations.** The panels represent the peptide (top panel), the HLA heavy α chain (middle panel), and β2-microglobulin (bottom panel). Error bars indicate the standard deviation.


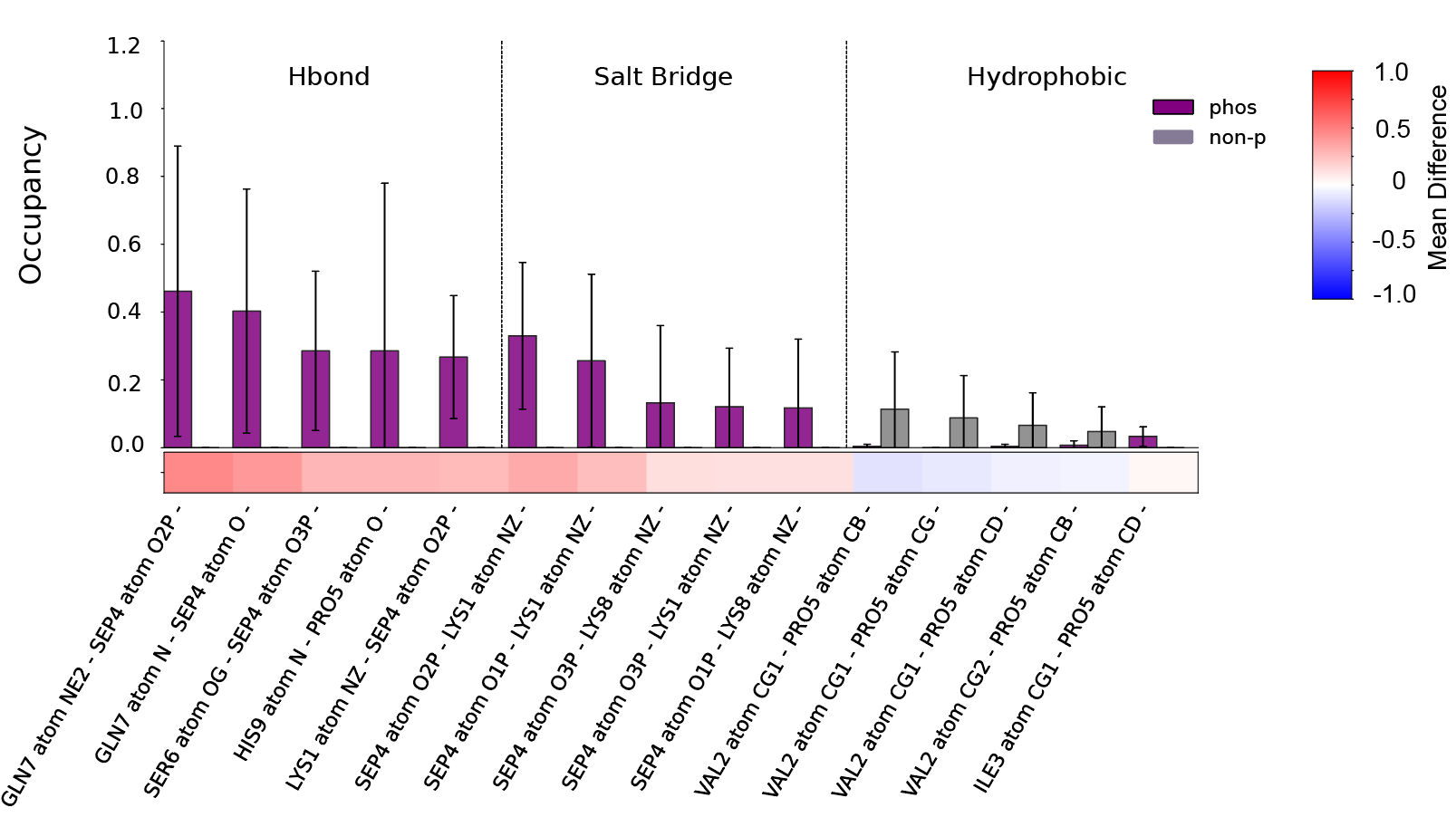


**Supplementary Figure 4. Noncovalent interaction differences between phosphorylated and non-phosphorylated solvated free peptides**. The color bar below each panel indicates the mean occupancy difference. Error bars indicate the standard deviation.


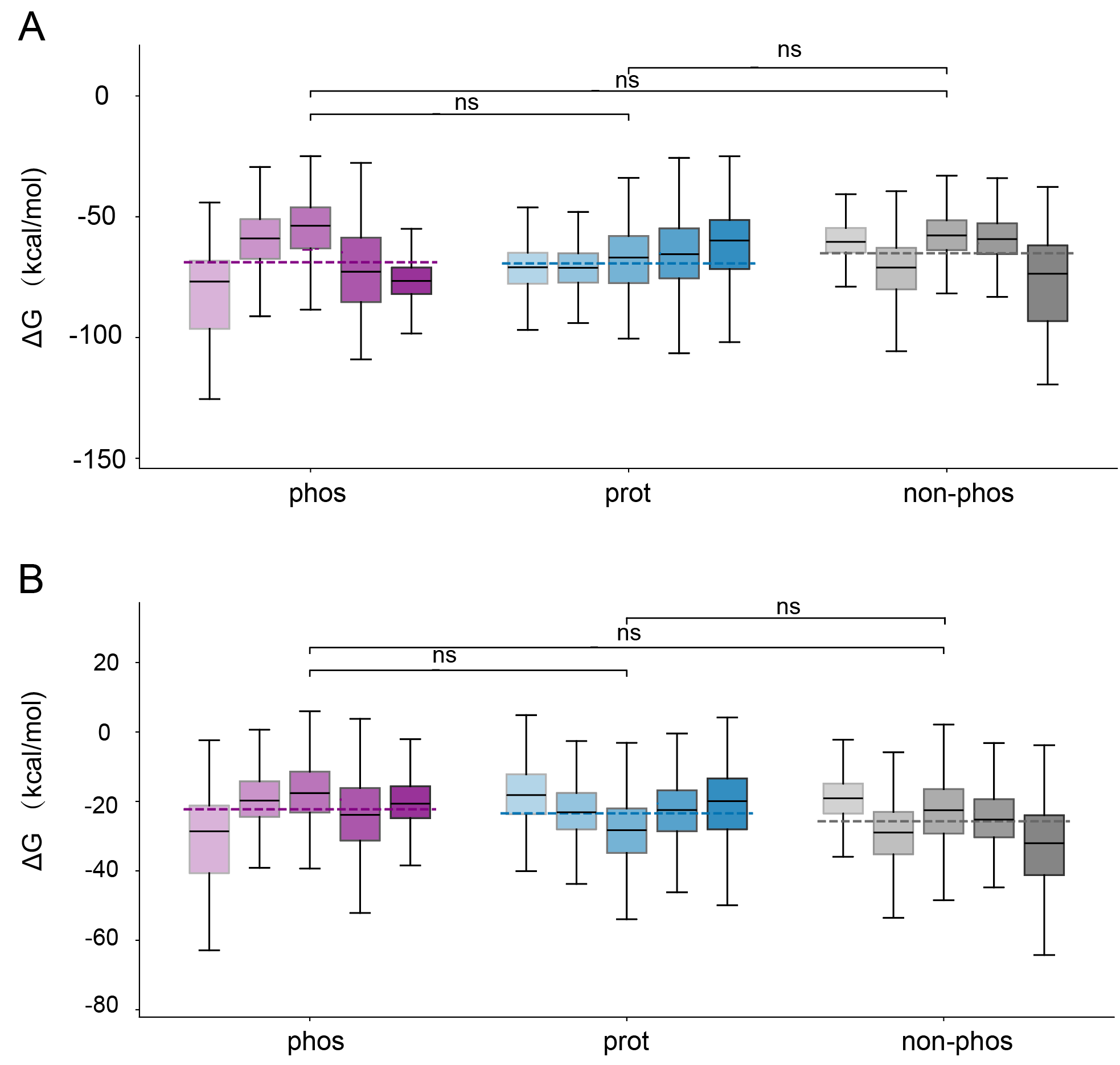


**Supplementary Figure 5. Binding free energy change calculated by GB and PB methods.** The free energy calculation results of phosphorylated (purple), protonated-phosphorylated (blue) and non-phosphorylated (grey) peptide-HLA systems calculated using the (A) GB and (B) PB methods, with statistical significance annotations. Dashed lines indicate the mean free-energy changes for each system.


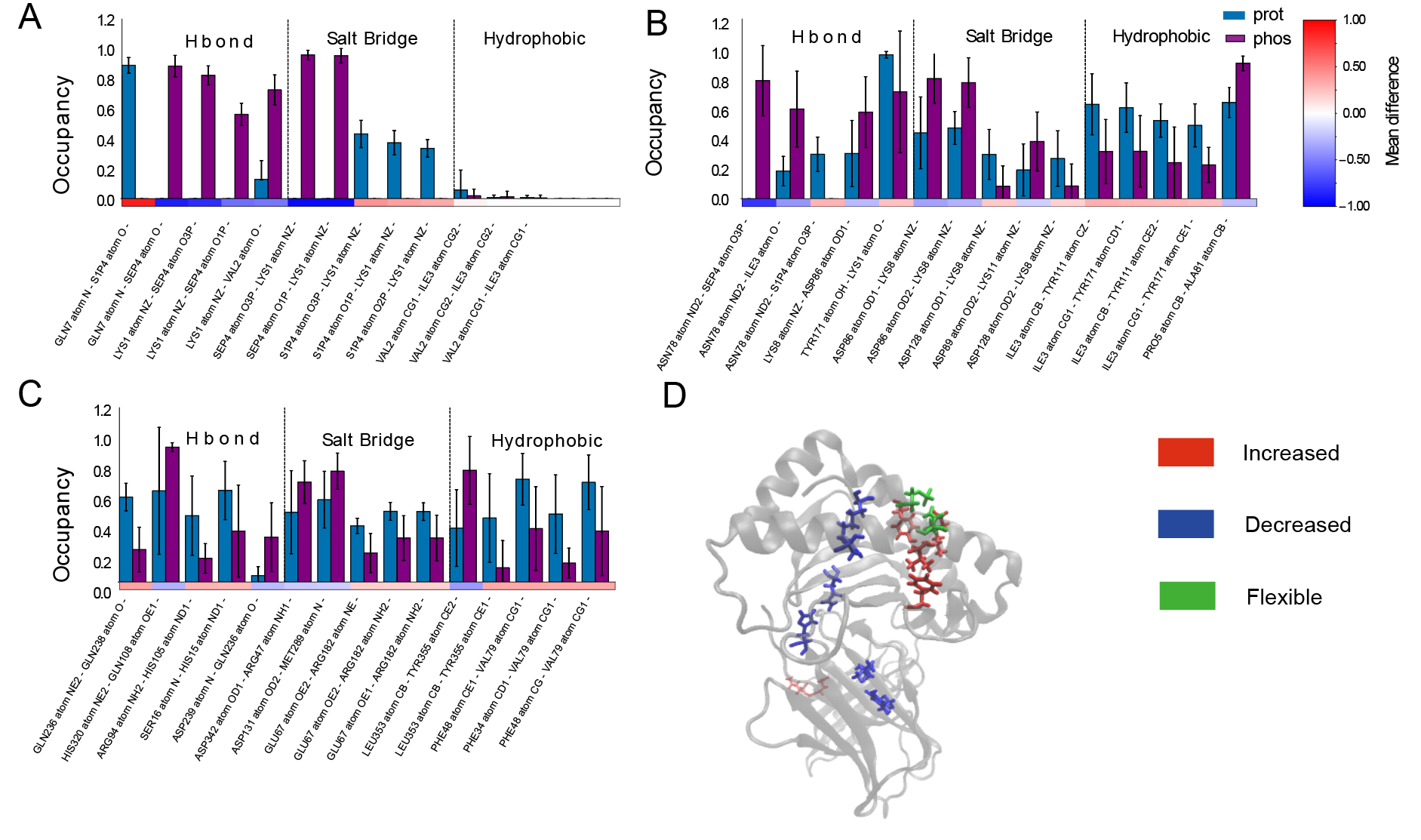


**Supplementary Figure 6. Noncovalent interaction differences between protonated-phosphorylated and non-protonated-phosphorylated peptide-HLA complexes.** (A) Peptide-peptide contacts, (B) peptide-HLA contacts and (C) HLA-HLA contacts. The color bar below each panel indicates the mean occupancy difference. (D) The typical residues involved in increased interaction (red), decreased interaction (blue) and flexible (green). If any residue is involved in both top increased and decreased interactions, we mark it as a flexible residue. Error bars indicate the standard deviation.
